# Serum Levels of MicroRNA-371a-3p (M371) Can Predict Absence or Presence of Vital Disease in Residual Masses After Chemotherapy of Metastatic Seminoma

**DOI:** 10.3389/fonc.2022.889624

**Published:** 2022-05-06

**Authors:** Klaus-Peter Dieckmann, Markus Klemke, Francesca Grobelny, Arlo Radtke, Inken Dralle-Filiz, Christian Wülfing, Gazanfer Belge

**Affiliations:** ^1^ Department of Urology, Asklepios Klinik Altona, Hamburg, Germany; ^2^ Department of Urology, Albertinen Krankenhaus, Hamburg, Germany; ^3^ Faculty of Biology & Chemistry, University of Bremen, Bremen, Germany; ^4^ mirdetect GmbH, Bremerhaven, Germany

**Keywords:** seminoma, metastases, chemotherapy, microRNA, M371, residual tumor, biomarker

## Abstract

**Background:**

Radiological evaluation of postchemotherapy residual masses of metastatic seminoma is characterized by poor diagnostic accuracy. Serum levels of microRNA-371a-3p (M371) involve high specificity and sensitivity for the primary diagnosis of seminoma. We evaluated if M371 levels can indicate the presence of vital disease in postchemotherapy residual masses in patients with metastatic seminoma.

**Methods:**

Twenty-three seminoma patients (median age 52 years) with residual masses had posttreatment measurements of serum M371 levels (group A), fourteen of whom had measurements also beforehand. The posttreatment results were compared with the clinical outcome during follow-up. Eleven patients with complete remission after treatment of metastatic seminoma (group B) and 33 men with non-malignant testicular diseases (group C) served as controls. M371 serum levels were measured by quantitative real-time PCR using miR-30b-5p as endogenous control. An evaluation was performed with descriptive statistical methods.

**Results:**

Twenty-two patients of Group A had uneventful follow-up so far, twenty-one of whom had M371 level <5, and one other had a mildly elevated level below relative quantity (RQ) = 10. One patient with a level of RQ = 26.2 rapidly progressed. The median posttreatment M371 level of the non-progressing patients of group A is not significantly different from the median level of the control group with complete remission (B). Before treatment, the median M371 levels in groups A and B were 507.6 and 143.9, respectively. In both groups, significant drops in M371 levels resulted from treatment.

**Conclusion:**

Normal M371 serum levels at the time of completion of treatment of metastatic seminoma indicate the absence of vital seminoma in residual masses, while elevated levels >RQ = 10 predict the presence of disease. The optimal timing of M371 measurement after chemotherapy and the appropriate cutoff level still need to be determined. Based on the present results, measuring serum M371 levels involves the potential of a novel tool for assessing postchemotherapy residual masses of metastatic seminoma.

## Introduction

**Figure 1:**
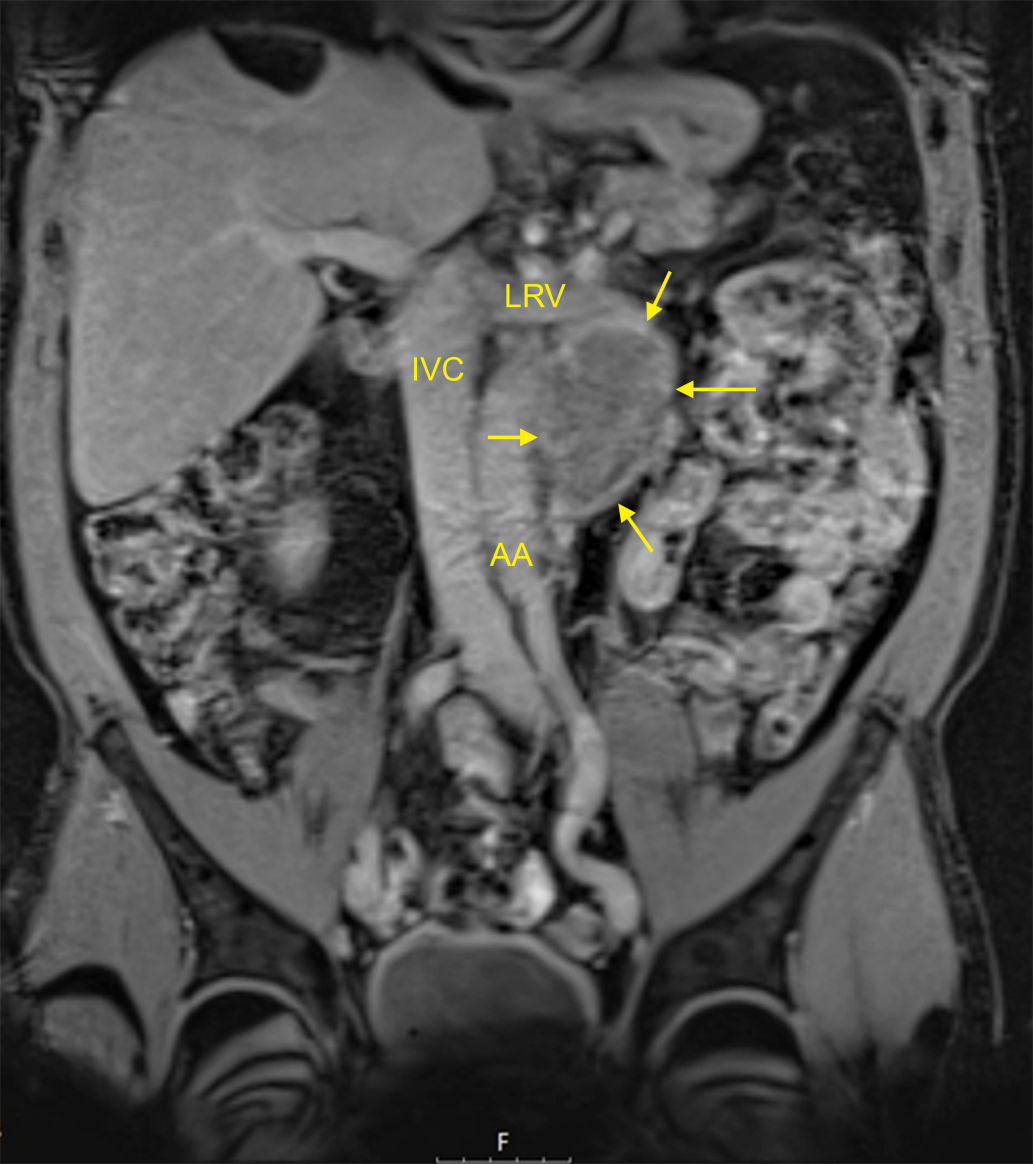
MRI of a typical residual mass resulting from chemotherapy of left-sided metastatic seminoma: 5 cm para-aortic mass (patient R8 in **Table 1**). T1-weighted imaging, fat-suppressed mode, coronal section. AA, abdominal aorta; IVC, inferior vena cava; LRV, left renal vein. Arrows denote the residual mass. This patient had an M371 level of relative quantity (RQ) = 1 and is continuously disease-free for 24 months. This figure illustrates that neither the imaging technique (i.e., MRI) nor the size of the residual mass (5 cm in this case) can safely predict the histology of the residual mass.

Cisplatin-based chemotherapy can provide a cure in the majority of patients with metastatic seminoma (1–3). Yet in about 73% of cases, radiological restaging will reveal only partial remission (PR) at the completion of treatment, which denotes a persisting mass of reduced size at the metastatic site (4). Most of these masses consist of necrosis or fibrosis, and only a few cases will still harbor vital seminoma (5, 6). CT and MRI cannot accurately identify those with vital seminoma (**Figure 1**). Likewise, serum tumor markers beta human chorionic gonadotropin (bHCG) and lactate dehydrogenase (LDH) are usually not useful in assessing residual masses of seminoma. Currently, clinical decision-making is based on the size of the residual mass. Surgical experience with residual tumors of seminoma had revealed that masses larger than 3 cm may contain vital disease in a substantial number of cases, while masses <3 cm usually consist of necrosis/fibrosis only (7, 8). Accordingly, current guidelines recommend surveillance of residual masses <3 cm (9–12). In larger masses, the traditional management is surgical resection. However, postchemotherapy resections of seminoma involved considerable morbidity, first, because seminoma patients are for the most part somewhat older than non-seminoma patients who usually tolerate retroperitoneal lymph node dissections (RPLNDs) without major problems. Second, postchemotherapy surgery in seminoma had been shown to be particularly tedious due to desmoplastic reactions around the great retroperitoneal vessels resulting in a substantial number of vascular complications (13–15). Therefore, surgery is no longer the option of choice in residual masses of seminoma sized >3 cm. Instead, guidelines currently recommend performing fluorodeoxyglucose PET/CT (FDG-PET/CT) to identify vital seminoma in the residual mass (16). However, a number of disadvantages have become obvious with this imaging modality. First, PET/CT scan involves more radiation dosage than normal CT. Second, it is rather cost-intensive, precluding widespread use. Third, PET/CT is only informative when performed not earlier than 6–8 weeks after completion of chemotherapy (17). Fourth and importantly, PET/CT scans involve a significant number of false-negative and false-positive readings (18–20). Above all, it remained unclear what kind of therapy should be instituted in the case of a positive PET/CT scan. Currently, a repeat scan is advocated in these cases with individual decisions to be taken in case of a positive repeat scan (12). Clearly, there is a need for better tools for assessing postchemotherapy residual masses of seminoma. Serum levels of microRNA-371a-3p (M371) have been shown to have a sensitivity of 90.1% and a specificity of 94.1% in the primary diagnosis of germ cell tumors (GCTs) (21–24). Moreover, M371 is still sensitive in small tumors. In residual masses of metastatic non-seminomatous tumors, the new marker has been shown to identify those with residual vital cancer (25, 26). However, the problem was that teratoma, which is present in about 30% of non-seminoma residual masses, does not express M371 (27–29). Thus, a negative M371 test in these cases will not obviate the need for postchemotherapy RPLND. In seminoma, there is no teratoma problem, and thus, the new marker could be particularly valuable in this histologic group. Therefore, the goal of the present study is to analyze the utility of serum levels of M371 for assessing the kind of postchemotherapy residual masses of seminoma. Our hypothesis is that M371 levels in the normal range will indicate the absence of vital seminoma in the residual mass, which will be proven by an uneventful later course. Conversely, an elevated M371 level will indicate the presence of vital seminoma, which will be evidenced by progressive disease.

## Patients and Methods

Twenty-three patients with residual masses subsequent to chemotherapy for metastatic seminoma (“group A”) underwent measurement of serum M371 levels at the time of completion of treatment. The median age of the patients is 52 years. Clinical stage (CS) at diagnosis according to the Lugano Classification was CS2b, CS2c, and CS3 in 5, 13, and 5 cases, respectively. CS3 was characterized by mediastinal and/or cervical lymph node metastases, and none had pulmonary metastases. With one exception, all of the patients belonged to the good prognosis group of the International Germ Cell Cancer Collaborative Group (IGCCCG) classification system. After orchiectomy with histological confirmation of seminoma, and serological confirmation of normal alpha-fetoprotein (AFP) levels, all patients underwent cisplatin-based chemotherapy according to current guideline recommendations. Modified management was applied to two patients. One received short-course radiotherapy subsequent to 2 cycles of PEB (cisplatin, etoposide, and bleomycin), because of severe gastrointestinal toxicity of PEB. The other patient received two courses of carboplatin monotherapy subsequent to two courses of PE due to critical myelodepression in the first two courses. After completion of chemotherapy, all patients had a measurement of serum levels of M371 and of classical tumor markers AFP, bHCG, and LDH after a median interval of 4 weeks (range 1–12 weeks) after the last application of chemotherapeutic drugs. Radiological re-staging revealed retroperitoneal residual masses sized 11–110 mm (**Figure 1**). One patient underwent postchemotherapy RPLND because of a positive PET/CT scan. None of the others received further therapy after the completion of primary treatment. Follow-up consisted of abdominal imaging with CT or MRI, along with chest X-ray, clinical examination, and tumor marker measurements according to national guidelines (9). Eleven patients had repeat measurements of M371 upon follow-up visits. The median time of follow-up is 18 months (2–51 months). We registered the results of follow-up examinations (no evidence of disease (NED) or progressive disease (PD)) and compared the clinical course with the result of the M371 measurement at the time of treatment completion.

Eleven seminoma patients served as controls, all of whom had achieved complete remission (CR) after treatment of metastatic disease; i.e., none had a residual mass larger than 1 cm (“group B”). The median age of these patients is 44 years. At diagnosis, CSs 2a, 2b, 2c, and CS3 were present in 1, 7, 1, and 2 patients, respectively. Seven of the controls underwent full-dose cisplatin-based chemotherapy, one patient received curative radiotherapy, and three had combined chemotherapy and radiotherapy. All had measurements of M371 at the completion of therapy, and all were followed up for a median interval of 18 months.

A second control group consisted of 33 patients with benign testicular diseases with a median age of 40 years (“group C”; details in **Supplementary Table**).

Pretreatment M371 serum levels (i.e., before chemotherapy) were measured in 14 patients of group A and in all of group B. Median pretreatment levels were compared to postchemotherapy levels in both groups. The median M371 levels of the CSs (CS2a/b, 2c, and 3) of combined groups (A and B) were compared to each other.

Serum samples for measurement of M371 were kept deep-frozen at −80°C until processing. Measurement results did not influence the clinical management of patients. Total RNA was isolated from 200 μl of serum using the miRNeasy Mini Kit (Qiagen, Hilden, Germany) according to the manufacturer’s instructions. Measurement of M371 was performed by quantitative real-time PCR as previously reported (22, 30) using the M371 test (mirdetect, Bremerhaven, Germany). Briefly, miR-30b-5p was used as endogenous control, and quantification of M371 was given as relative quantity (RQ) in relation to miR-30b-5p. The relative expression of miR-371a-3p was calculated according to the comparative ΔCT method (31). RQ ≤ 5 was considered a normal range or non-expression of the miR. The classical tumor markers were measured in the routine hospital laboratory according to institutional standard operating procedures.

Descriptive statistical methods were used for the analysis of data. The M371 expression of the various groups is presented as median with interquartile ranges (IQRs). The statistical evaluation was carried out with SPSS version 26 (IBM, Armonk, NY, USA). For the analysis of differences between two independent variables, the Mann–Whitney U test was used. Significance was assumed at p < 0.05.

Ethical approval was provided by the Ethical committee of Ärztekammer Hamburg (MC 152/19, July 15, 2019). All study activities were conducted according to the Declaration of Helsinki of the World Medical Association as amended by the 64th General Assembly, October 2013.

## Results

**Figure 2:**
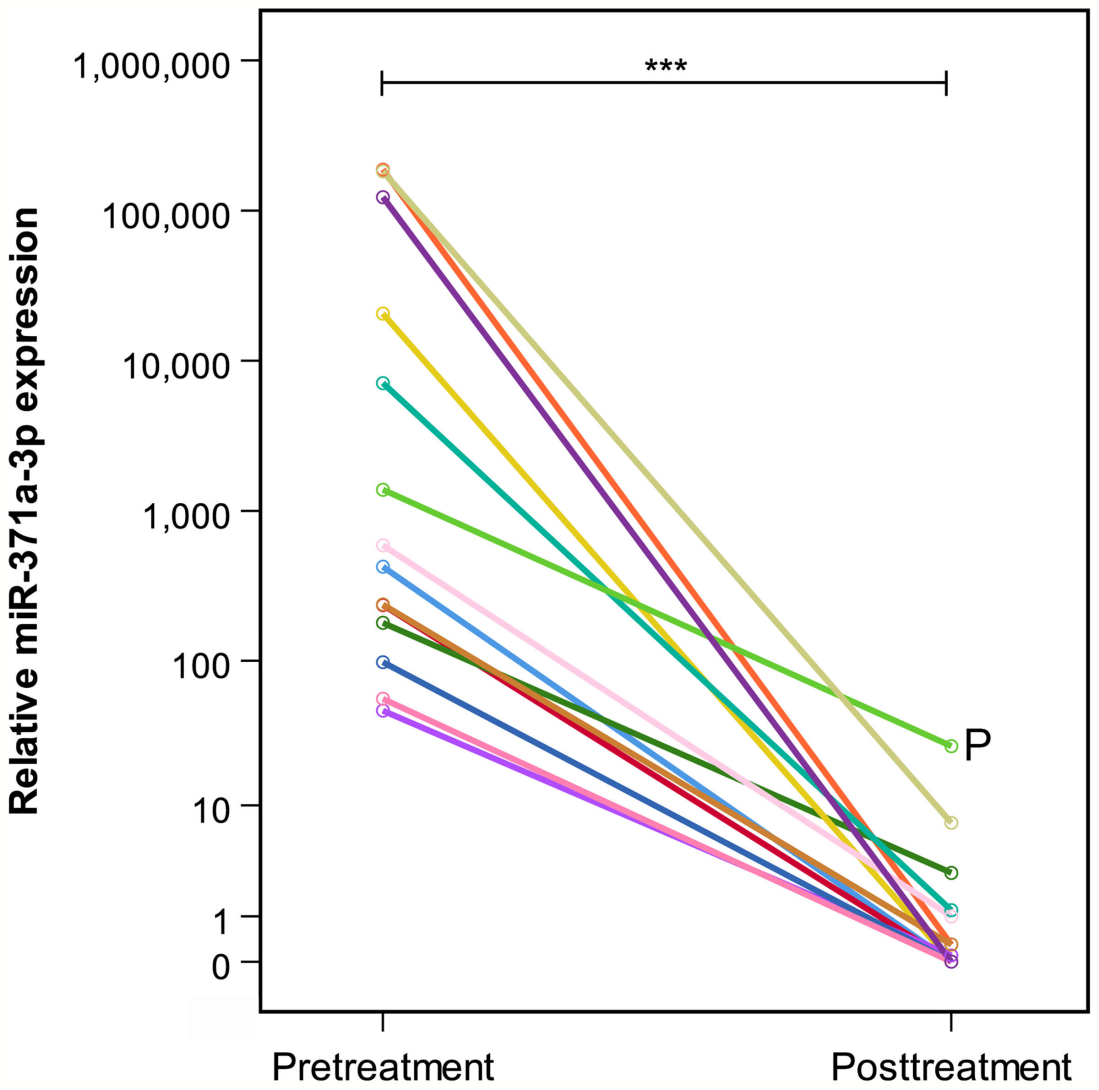
Comparison of individual prechemotherapy and postchemotherapy M371 expression levels in 14 patients with residual tumors (group A). All patients but one had decreased to levels below relative quantity (RQ) = 10. P indicates patient who developed progressive seminoma. He failed to have a decrease of below RQ = 10. The y-axis is plotted on a logarithmic scale. ***p ≤ 0.001.

**Figure 3:**
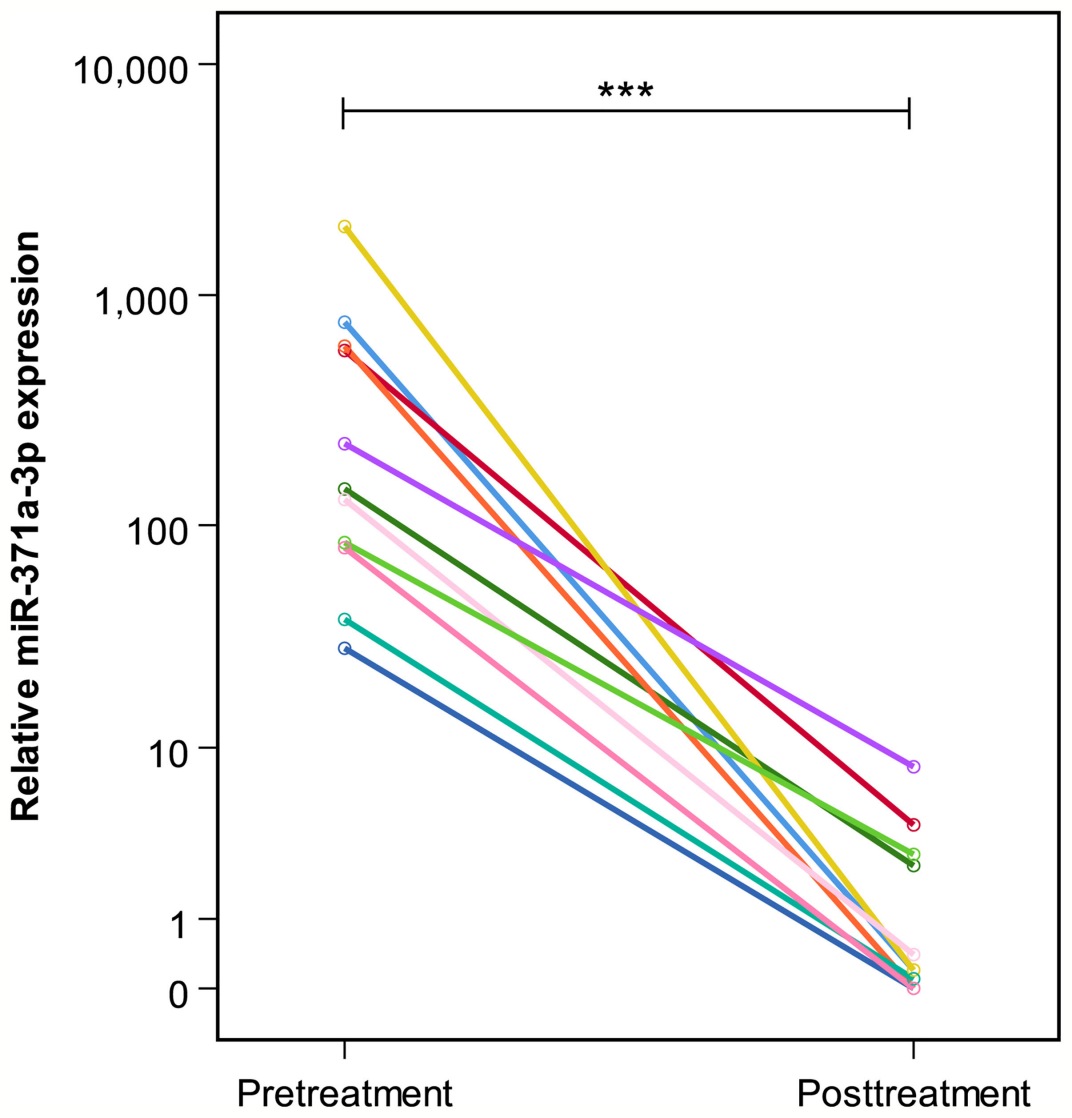
Pre- and posttreatment M371 levels in 11 patients with complete remission (group B). The y-axis is plotted on a logarithmic scale. ***p ≤ 0.001.

**Figure 4:**
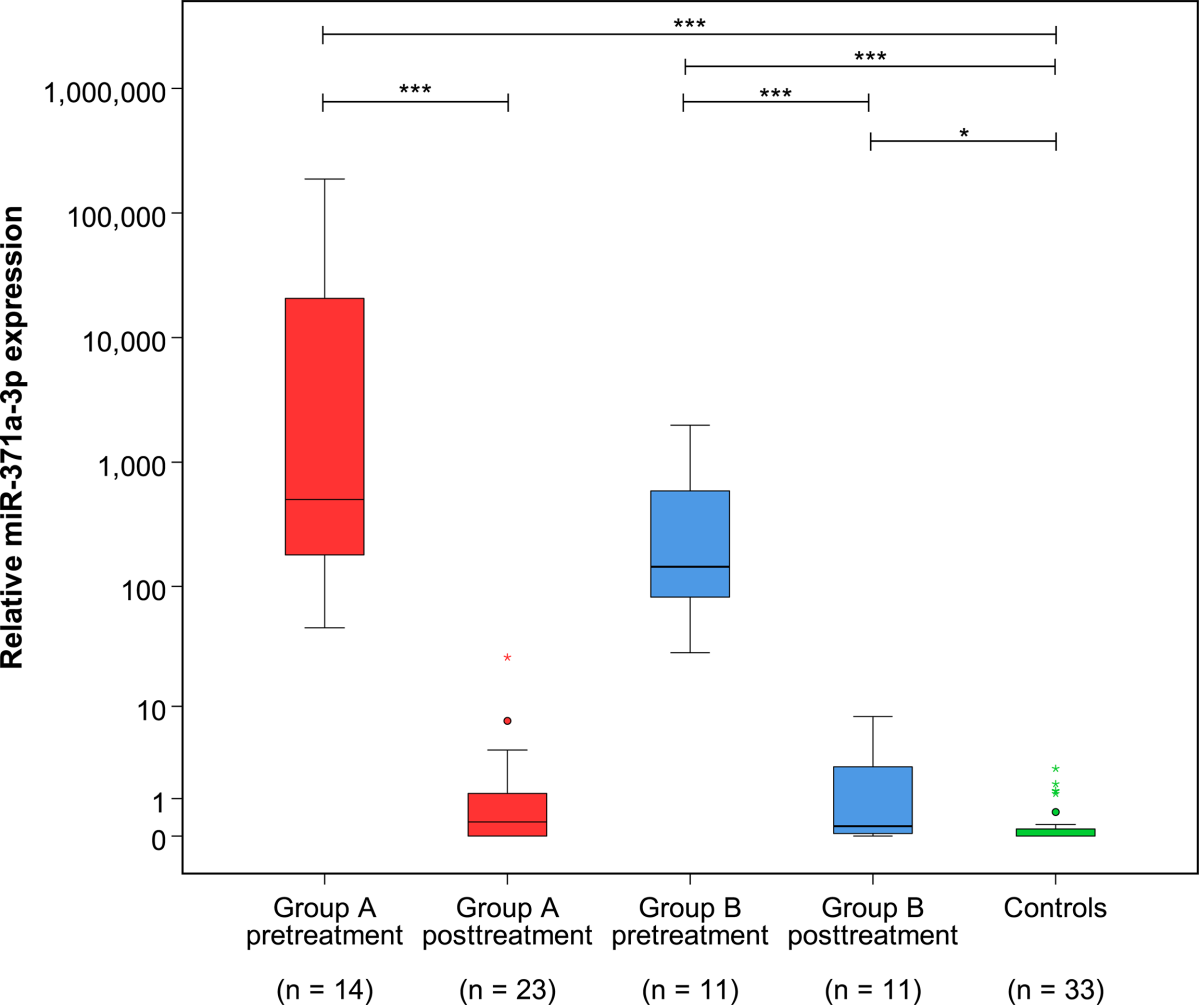
Relative M371 expression in serum of patients with residual tumors (group A) and complete remission (group B) pre- and posttreatment as well as in controls (group C). Boxplots indicate the median relative M371 expressions with interquartile ranges in the five groups. The median M371 expressions in group A and B pretreatment are significantly higher than posttreatment levels and the expression in controls (***p ≤ 0.001). The median posttreatment M371 expressions in group B are significantly different from those of the non-malignant control group (*p ≤ 0.05), but there is no significant difference between the posttreatment levels of groups A and B. The outlier with the highest expression in group A posttreatment is the case that developed progressive seminoma. The y-axis is plotted on a logarithmic scale.

**Figure 5:**
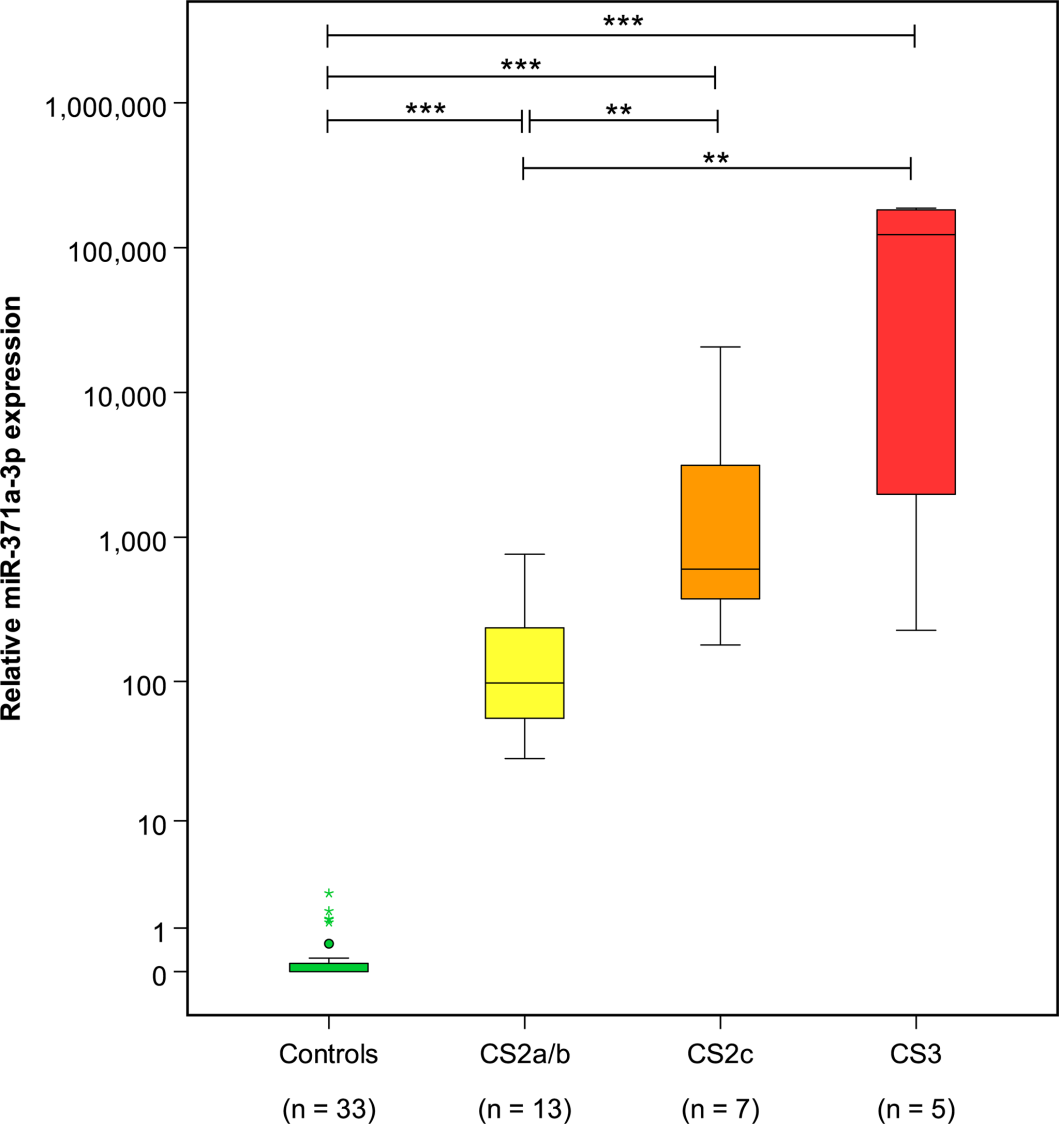
Pretreatment M371 levels in the various clinical stages of seminoma and the control group. Boxplots indicate the median relative M371 expression with interquartile ranges in the clinical stages CS2a/b, CS2c, and CS3. The median M371 expression in the clinical stage CS2a/b is significantly lower than the expressions in CS2c and CS3 (**p ≤ 0.01). Pretreatment median levels of all three clinical stages are significantly different from those of controls (***p ≤ 0.001). The y-axis is plotted on a logarithmic scale.

At the completion of therapy, all patients (groups A and B) had normal serum levels of bHCG and LDH. Individual results of posttreatment M371 measurements along with individual time intervals from the end of treatment to blood sampling are listed in **Table 1**. One patient with clearly elevated M371 level after completion of chemotherapy (RQ = 26.2) developed progression 3 months later as evidenced by positive PET/CT finding and rising bHCG level. During the same time interval, the M371 level increased to RQ = 1834.9 in this patient. He was cured with high-dose chemotherapy and subsequent surgery. One patient had a marginally elevated level (RQ = 7.4) above the cutoff of RQ = 5, which is the level that was found to separate expression from non-expression of the marker in our previous studies. However, he fared well so far without progression. One patient with a postchemotherapy M371 level of zero (RQ = 0) underwent RPLND of the residual tumor. Histology of the surgical specimen revealed necrosis and fibrosis only. All other patients had M371 levels below RQ = 5, and all are well with no evidence of disease after a median time of follow-up of 18 months. Ten patients had repeat M371 measurements during follow-up; all were below the cutoff level, mirroring the disease-free course of these patients. **Table 2** lists the patients with CR after treatment, one of whom had an M371 level of 8.1 at the completion of therapy. He is well with no evidence of disease at 6 months of follow-up. All others had RQ levels below the cutoff of 5, and all of them are well, with no evidence of disease after a median time of follow-up of 18 months. **Figure 2** shows the decreases of M371 levels in individual patients with residual masses after chemotherapy (group A). The decreases in M371 levels in individual patients with CR are shown in **Figure 3** (group B). **Figure 4** shows the median M371 levels before and after treatment along with the median M371 level of controls (group C). Expectedly, there were clearly elevated pretreatment M371 levels in both seminoma groups (group A, RQ = 507.6, IQR = 158.9–46,172.3; group B, RQ = 143.9, IQR = 79.7–600.5), and in both groups, there was a significant drop of median levels after completion of treatment (group A, RQ = 0.3, IQR = 0.0–1.2; group B, RQ = 0.2, IQR = 0.0–2.8). The pretreatment levels in groups A and B were significantly higher than those of controls (group C) (p = 2.93 × 10^−12^ and p = 1.30 × 10^−10^, respectively) (**Supplementary Table**). Posttreatment levels of groups A and B are not significantly different from each other (p = 0.581), whereas the difference between the posttreatment levels of group B and the non-malignant controls (group C) is slightly significant (p = 0.025). However, all median values of groups A (posttreatment), B (posttreatment), and C (non-malignant controls) are below the cutoff of RQ = 5. **Figure 5** shows the median pretreatment M371 levels in the various CSs of combined groups A and B. Median expression levels in the CS2a/b, CS2c, and CS3 were RQ = 97.6 (IQR = 50.6–330.3), RQ = 600.5 (IQR = 237.5–7,107.1), and RQ = 122,868.6 (IQR = 1,103.0–184,888.9), respectively. Significant differences were observed between CS2a/b and CS3 (p = 0.003) and between CS2a/b and CS2c (p = 0.002), while the difference between CS2c and CS3 was not significant (p = 0.149).

**Table 1:** Clinical data and relative M371 expression in serum of seminoma patients with residual tumors after chemotherapy or radiotherapy (group A).

Patient ID	Age (years)	Size of the residual tumor (mm)	Primary CS	Type of chemotherapy	Follow-up	RQ M371 before chemotherapy	RQ M371 after chemotherapy	Time interval to blood sampling (weeks)^+^
R1	46	55	2c	3×PEB	Necrosis	–	0	8
R2	35	15	2b	3×PEB	NED 12 M	424.8	0	4
R3	67	11	2b	2×PEB+Rx	NED 39 M	235.8	0	4
R4	31	25	2c	3×PEI	NED 46 M	179.3	2.9	2
R5	52	90	3, interm.	4×PEB	NED 24 M	187,436.2	0.3	4
R6	46	11	2c	3×PEB	NED 15 M	20,606.8	0	4
R7	61	40	2c	2×PE+2×Carbo	NED 36 M	7,107.1	1.2	6
R8	53	50	2c	4×PE	NED 24 M	590,3	1	3
R9	47	25	2b	4×PE	NED 18 M	45.9	0.1	1
R10^*^	47	50	2c	4×PEI	Progression 3M	1,384.5	26.2	2
R11	40	15	2b	3×PEB	NED 18 M	97.6	0	3
R12	54	30	2c	4×PE	NED 18 M	–	0.8	6
R13	45	29	2c	3×PEB	NED 18 M	–	0	12
R14	41	40	2c	4×PE	NED 24 M	–	0	8
R15	53	110	2c	4×PEB	NED 51 M	–	0.7	12
R16	53	60	3	4×PEB	NED 24 M	–	1.6	4
R17	37	44	2c	3×PEB	NED 6 M	–	1.2	12
R18	52	20	2b	4×PE	NED 6 M	55.2	0	2
R19	63	32	3	4×PEB	NED 9 M	182,341.5	7.4	2
R20	55	30	2c	3×PEB	NED 48 M	–	0	8
R21	54	12	3	4×PEB	NED 36 M	–	3.9	8
R22	66	17	2c	4×PE	NED 3 M	237.5	0.3	2
R23	29	36	3	4×PEB	NED 2 M	122,868.6	0	1

CS, clinical stage (according to Lugano classification); Carbo, carboplatin monotherapy; interm., intermediate prognosis according to International Germ Cell Cancer Collaborative Group (IGCCCG); M, month; –, no serum samples available; NED, no evidence of disease; PE, cisplatin and etoposide; PEB, cisplatin, etoposide, and bleomycin; PEI, cisplatin, etoposide, and ifosfamide; Rx, radiotherapy; RQ, relative quantity.

^*^Patient developed a progressive seminoma; the RQ was 1,834.9 after 3 months.

^+^Median interval to first blood sampling is 4 weeks (range 1–12 weeks).

**Table 2:** Clinical data and relative M371 expression in serum of seminoma patients with complete remission after chemotherapy or radiotherapy (no residual tumors, group B).

Patient ID	Age (years)	Primary CS	Chemotherapy	RQ M371 before chemotherapy	RQ M371 after chemotherapy
T1	26	2b	3×PEB	762.1	0.2
T2	38	2b	3×PEB	574.9	4.1
T3	51	2b	Rx	143.9	2.4
T4	48	2c	3×PEB	600.5	0
T5	43	3	4×TIP	1,979.4	0.2
T6	43	2b	4×PE	38.5	0.1
T7	49	2b	1×PE+Rx	129.4	0.4
T8	47	3	4×PE	226.6	8.1
T9	44	2b	3×PEB	83.9	2.8
T10	30	2a	Carbo+Rx	28.6	0
T11	45	2b	PE+Rx	79.7	0

CS, clinical stage (according to Lugano classification); Carbo, carboplatin monotherapy; PE, cisplatin and etoposide; PEB, cisplatin, etoposide, and bleomycin; Rx, radiotherapy; RQ, relative quantity; TIP, paclitaxel, ifosfamide, and cisplatin.

## Discussion

The central result of the present study is that normal or slightly elevated M371 serum levels (RQ < 10) at the time of completion of treatment are associated with disease-free status in patients undergoing chemotherapy for metastatic seminoma. Importantly, the one patient who developed progressive disease was shown to have a distinctly elevated M371 level at the end of chemotherapy (RQ = 26.2). Clearly, as this latter observation is still a single finding, interpretations must be drawn cautiously. The median M371 serum level of the patients who remained disease-free despite residual masses after treatment of metastatic seminoma was not significantly different from the median level of seminoma patients achieving CR after treatment of metastatic seminoma, and both median levels were below the cutoff of RQ = 5. Thus, the present study provides the first piece of evidence for the understanding that normal M371 levels at the end of chemotherapy for metastatic seminoma do likely indicate the absence of viable residual seminoma. Noteworthy, the median M371 level of the healthy controls is significantly lower than the postchemotherapy median level of group B. However, this statistical finding is probably not relevant, clinically, because first, both of the two median levels are clearly below RQ = 5, and generally, M371 levels below that cutoff are considered as non-expression of the marker. Second, the differences are truly minor and may represent biological chance scatterings of M371 expressions as well as technical chance events upon miR measurements.

Our results suggest that M371 levels could in fact be used as a diagnostic tool for assessing residual masses after chemotherapy of seminoma. Presently, the cutoff value is not clear, since one patient with residual tumor and one of those with CR had slightly elevated levels in the RQ range of 5–10. As both patients experienced an uneventful clinical course, one could speculate that the cutoff level for assessing residual tumors of seminoma should be set somewhat higher than the cutoff of RQ = 5 for primary diagnosis of GCTs, probably in the range of 8–10. Noteworthy, as the two patients with slightly elevated levels were examined only shortly after completion of chemotherapy, it appears conceivable that further decreases of the M371 levels might occur during the following weeks after cessation of treatment. Moreover, both patients had CS3 disease with huge tumor masses at the time of diagnosis. It is known from previous studies on monitoring M371 levels during the courses of chemotherapy that CS3 patients had much slower decays of serum levels than patients with lower stages (22, 32, 33). It is thus conceivable that M371 levels could have further dropped during the later course. Unfortunately, repeat measurements are not available in these two patients. The hypothesis that further decreases in M371 level may occur during the weeks after chemotherapy mirrors the experience with PET/CT scans that should be performed not earlier than 6 weeks after completion of treatment because early scans may give rise to false-positive results. Accordingly, repeat PET/CT scans have shown that originally positive scans can turn negative after an interval of 6–8 weeks (20, 34).

Another noteworthy result of this study is the association of median M371 levels with CSs. Significant differences in median M371 levels between seminoma stages CS1, CS2, and CS3 had been reported earlier (22, 33, 35, 36). However, the present evaluation involves a more granular analysis of the CS2 substages. The results are in accord with the view that there is an association between serum M371 levels and tumor bulk, which in turn underscores the usefulness of this microRNA as a valuable biomarker for GCTs.

The limitations of the present study relate to the small sample size, and it must be emphasized that only one case of the present series had disease progression. One other limitation is probably the lack of postchemotherapy PET/CT examinations in the majority of cases. However, the rather unequivocal results in the patients without progression may still lend credit to the conclusions drawn. Some uncertainty may result from the shorter than 1 year of follow-up interval in five patients. Finally, the lack of repeat measurements at least in the two patients with slightly elevated levels must be considered a limitation.

In residual masses resulting from chemotherapy of non-seminoma, elevated M371 levels had been shown to denote the presence of vital germ cell cancer (25). However, a normal M371 test does not justify the omission of postchemotherapy surgery, because teratoma could still be present. This particular subtype of GCT is found in about 30% of non-seminomatous residual tumors, and it definitely requires excision (37, 38). Unfortunately, this subtype does not express M371. As seminoma does not contain teratoma elements, seminomatous residual masses can be assessed much more accurately by measurement of M371 than their non-seminomatous counterparts. In conclusion, measuring M371 levels could significantly simplify clinical decision-making in seminoma patients with residual masses after treatment of metastatic disease. If the present data are confirmed in a larger patient population, M371 measurements will probably obviate the use of PET/CT scans for assessing residual masses after treatment of metastatic seminoma. Also, adjunctive treatment measures could be tailored to patients with persisting elevated M371 levels.

## Data Availability Statement

The original contributions presented in the study are included in the article/**Supplementary Material**, further inquiries can be directed to the corresponding author/s.

## Ethics Statement

The studies involving human participants were reviewed and approved by the Ethical committee of Ärztekammer Hamburg (MC 152/19, July 15, 2019). The patients/participants provided their written informed consent to participate in this study.

## Author Contributions

K-PD and GB conceived the study. K-PD and CW supervised the whole project. MK, ID-F, and FG performed the data curation. AR, MK, FG, and GB performed the statistical analysis. K-PD and GB wrote the manuscript. CW, MK, and AR participated in the manuscript editing and discussion. All authors listed have made a substantial, direct, and intellectual contribution to the work and approved it for publication.

## Funding

This investigation was supported by prize money awarded to K-PD with the Maximilian Nitze Preis der Deutschen Gesellschaft für Urologie in 2019.

## Author Disclaimer

All claims expressed in this article are solely those of the authors and do not necessarily represent those of their affiliated organizations, or those of the publisher, the editors, and the reviewers. Any product that may be evaluated in this article, or claim that may be made by its manufacturer, is not guaranteed or endorsed by the publisher.

## Conflict of Interest

K-PD and GB declare ownership shares of each 9.71% of mirdetect GmbH, Bremerhaven, Germany. AR is an employee of mirdetect, GmbH, Bremerhaven, Germany.

The remaining authors declare that the research was conducted in the absence of any commercial or financial relationships that could be construed as a potential conflict of interest.

## Publisher’s Note

All claims expressed in this article are solely those of the authors and do not necessarily represent those of their affiliated organizations, or those of the publisher, the editors and the reviewers. Any product that may be evaluated in this article, or claim that may be made by its manufacturer, is not guaranteed or endorsed by the publisher.
