# Supplementary material for: Serum Levels of MicroRNA-371a-3p (M371) Can Predict Absence or Presence of Vital Disease in Residual Masses After Chemotherapy of Metastatic Seminoma
Source: Front Oncol. 2022 May 6;12:889624. doi: 10.3389/fonc.2022.889624 (PMC9121896; doi:10.3389/fonc.2022.889624)
Supplement: Supplementary file 1 [file Table_1.docx]

**Supplementary table.** Clinical data and relative M371 expression of the control samples from patients with other testicular diseases with indication of diagnosis and patient age (group C).

| Patient ID | | Age (years) | Diagnosis | RQ M371 | |
| --- | --- | --- | --- | --- | --- |
| C1 | 60 | | Epididymitis | | 0.15 |
| C2 | 19 | | Epididymitis | | 0 |
| C3 | 73 | | Epididymitis | | 0.05 |
| C4 | 18 | | Varicocele | | 0.07 |
| C5 | 38 | | Spermatocele | | 1.62 |
| C6 | 41 | | Epididymo-orchitis | | 0 |
| C7 | 29 | | Testicular epidermoid cyst | | 0 |
| C8 | 55 | | Orchitis | | 1.32 |
| C9 | 57 | | Testicular pain | | 0.01 |
| C10 | 57 | | Testicular atrophy | | 0 |
| C11 | 69 | | Hydrocele | | 0 |
| C12 | 51 | | Infertility | | 0.01 |
| C13 | 38 | | Spermatocele | | 0.01 |
| C14 | 18 | | Testicular torsion | | 0 |
| C15 | 25 | | Urethritis | | 0.01 |
| C16 | 51 | | Testicular fibrosis | | 0 |
| C17 | 70 | | Hydrocele | | 2.48 |
| C18 | 53 | | Leydig cell hyperplasia | | 0.24 |
| C19 | 48 | | Orchitis | | 0 |
| C20 | 38 | | Chronic testicular pain | | 0 |
| C21 | 24 | | Chronic testicular pain | | 0 |
| C22 | 30 | | Epididymitis | | 0.04 |
| C23 | 59 | | Orchialgia | | 0 |
| C24 | 57 | | Epididymitis | | 0.14 |
| C25 | 40 | | Granulomatous orchitis | | 0 |
| C26 | 30 | | Hydrocele | | 0 |
| C27 | 18 | | Orchialgia | | 0 |
| C28 | 58 | | Orchialgia | | 0 |
| C29 | 38 | | Erectile dysfunction | | 1.19 |
| C30 | 26 | | Testicular torsion | | 0 |
| C31 | 29 | | Epididymitis | | 1.29 |
| C32 | 30 | | Benign epididymal tumor | | 0 |
| C33 | 52 | | Orchialgia | | 0.56 |

RQ: Relative quantity.
